# Irreversible electroporation for prostate cancer: another promising focal therapy

**DOI:** 10.31744/einstein_journal/2024RC0779

**Published:** 2024-10-23

**Authors:** Bruno Pagnin Schmid, Guilherme Cayres Mariotti, Guilherme Marcelino de Miranda, Rodrigo Gobbo Garcia, Oskar Kaufmann

**Affiliations:** 1 Hospital Israelita Albert Einstein Department of Interventional Radiology São Paulo SP Brazil Department of Interventional Radiology, Hospital Israelita Albert Einstein, São Paulo, SP, Brazil.; 2 Hospital Israelita Albert Einstein Department of Urology São Paulo SP Brazil Department of Urology, Hospital Israelita Albert Einstein, São Paulo, SP, Brazil.

**Keywords:** Prostatic neoplasms, Irreversible electroporation therapy, Radiology, interventional, Ablation techniques, Multiparametric magnetic resonance imaging

## Abstract

Radical treatment for prostate cancer is associated with significant morbidity. Percutaneous image-guided irreversible electroporation is a non-thermal ablative technique that has emerged as a valuable option. This study describes the case of a patient with prostate cancer who was successfully treated using irreversible electroporation. We report the case of a 72-year-old male patient who presented with elevated PSA (4.0ng/mL) during routine testing. Multiparametric magnetic resonance imaging of the prostate revealed a 0.8 cm lesion in the posterolateral aspect of the right midgland with marked hypointensity on ADC (ACR PI-RADS 4). The transperineal prostate revealed acinar adenocarcinoma (Gleason Score 3+3=6; International Society of Urological Pathology=1). Serum PSA levels reduced to 1.04ng/mL 32 days after the procedure and remained within normal limits (1.26ng/mL) after 349 days. Follow-up imaging performed 90 days later with prostate-specific membrane antigen PET/MRI showed size reduction, retraction, and diffuse hypointensity in the peripheral zone of the right prostate lobe, with no increase in prostate-specific membrane antigen uptake. Magnetic resonance imaging found no suspicious lesions 367 days after irreversible electroporation. At the final clinical follow-up at 390 days, the patient was asymptomatic. Our findings illustrate the potential of irreversible electroporation as a possible alternative treatment for prostate cancer.

## INTRODUCTION

Radical treatment for prostate cancer (PC) is associated with significant morbidities, including incontinence and erectile dysfunction.^(1)^ Focal treatments with a lower complication profile, such as high-intensity focal ultrasound (HIFU) and focal cryotherapy, are other attractive options.^(1)^ However, these methods carry the risk of thermal lesions that can significantly impact the patient's quality of life.

Percutaneous image-guided irreversible electroporation (IRE) is a non-thermal ablative technique based on the generation of high-voltage electrical pulses between needles that induces programmed cell death by creating nanopores in the cellular membrane.^(2,3)^ Irreversible electroporation has produced promising results in multiple types of cancers and could be replicated in PC.^(3,4)^ In this report, we describe the case of a patient with PC who was successfully treated with IRE.

## CASE REPORT

**Figure 1:**
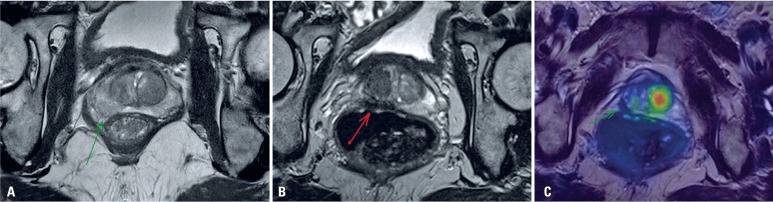
Imaging findings. Magnetic resonance imaging using a T2 weighted sequence shows (A) a slightly hypointense lesion on the right midgland. (B) a markedly hypointense region after treatment on T2-weighted sequence, and (C) no increased uptake of prostate-specific membrane antigen on PET/MRI after treatment.

A 72-year-old male patient presented with elevated PSA (4.0ng/mL) during routine testing. To investigate this slightly increased PSA value, multiparametric magnetic resonance imaging (MRI) of the prostate was performed, which revealed a 0.8cm ill-defined lesion in the posterolateral aspect of the right midgland with marked hypointensity on ADC (ACR PI-RADS 4) (Figure 1). The patient was otherwise healthy, with no relevant medical history.

**Figure 2:**
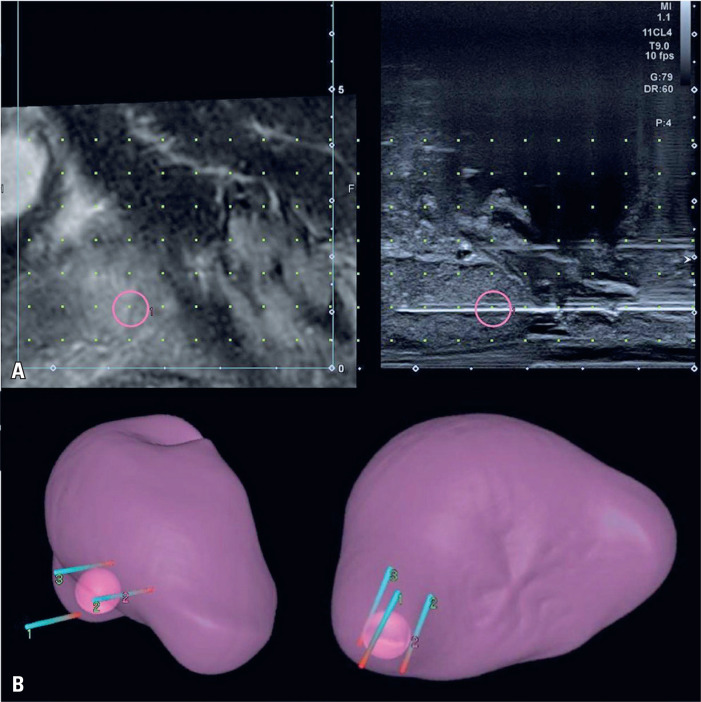
Transperineal biopsy. A) A real-time fusion of the prostate MRI coupled with the sagittal view of the transrectal ultrasound during the transperineal biopsy of the nodule. B) A 3D reconstruction depicts the position of the electroporation probes relative to the prostatic nodule

Next, a transperineal prostate biopsy was performed, and as per institutional protocol, 18 random samples and four MRI-targeted samples were obtained (Figure 2). The resulting analysis showed that only one of these prostate specimens was positive for acinar adenocarcinoma (Gleason Score 3+3=6; International Society of Urological Pathology=1).

The risks and benefits associated with all the management options were discussed with the patient. He felt that prostatectomy at this point was too radical for what seemed to be a rather unaggressive/early-stage tumor and would entail complications that he was not willing to undertake. He was also not in favor of a watchful waiting approach. Hence, after discussing local therapies, IRE ablation was chosen as the treatment method.

The procedure was performed at the Interventional Medicine Center of a quaternary institution under general anesthesia, using 2g of Ceftriaxone as antibacterial prophylaxis. The patient was placed in the lithotomy position, and the scrotum was elevated and held out of the way using tape to expose the perineum, which was prepared with chlorhexidine solution.

The IRE was performed using the NanoKnife® system (AngioDynamics, Queensbury, NY, USA) and three 15cm x 19G electrodes (NanoKnife, AngioDynamics, Latham, NY, USA) with an exposure of 4cm surrounding the lesion (Figure 2). A biplanar endorectal ultrasound probe (6–12 MHz, Canon Aplio a, Otawara, Japan) coupled with real-time fusion of the previous MRI was used to guide probe insertion. A 10-pulse test of this setup was carried out to adjust the voltage between the probes, and then two therapeutic cycles of 80-pulse each were performed to obtain a current of over 20 A between the probes. No complications were observed.

Thirty-two days after the procedure, serum PSA levels reduced to 1.04ng/mL and remained within normal limits (1.26ng/mL) after 349 days. Follow-up imaging performed after 90 days with prostate-specific membrane antigen (PSMA) PET/MRI (Figure 1) showed size reduction, retraction, and diffuse hypointensity in the peripheral zone of the right prostate lobe, with no increased PSMA uptake.

**Figure 3:**
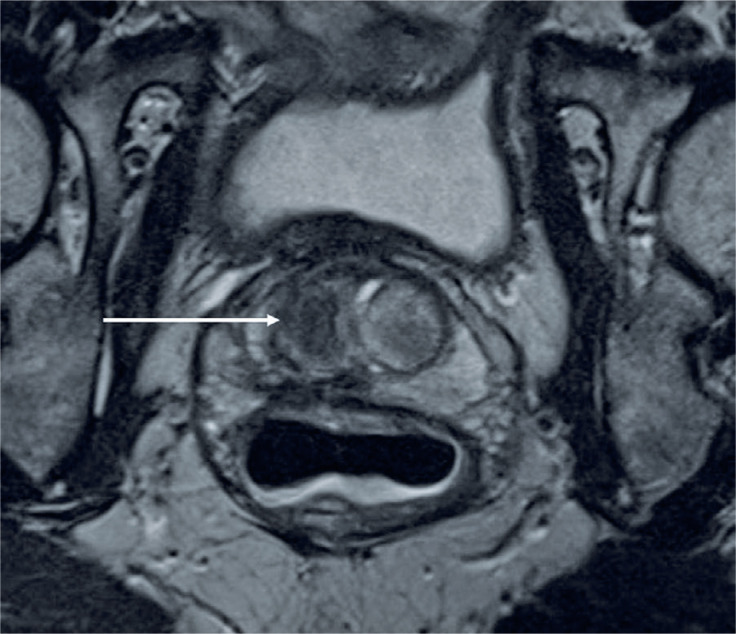
Magnetic resonance imaging (T2-weighted sequence) performed 367 days after irreversible electroporation shows no suspicious lesions. The ablation area appears with marked hypointensity (white arrow)

An MRI performed 367 days after IRE revealed no suspicious lesions (Figure 3). At the final clinical follow-up at 390 days, the patient was asymptomatic.

This study was approved by the Ethics Committee of *Hospital Israelita Albert Einstein* (CAAE: 70255123800000071; #6.188.913).

## DISCUSSION

This study illustrates that IRE is an effective alternative treatment for PC.

A recent prospective study evaluated the oncological and quality-of-life outcomes following focal irreversible electroporation as primary treatment for localized prostate cancer in 123 patients.^(5)^ During a median follow-up of 36 months, no residual disease was seen in the control biopsy in 90.2%-97.3% of patients, with metastasis-free survival of 99% and overall survival of 100%.^(5)^ Another study, including 63 patients with organ-confined clinically significant PC with a minimum of six months follow-up, showed in-field and whole-gland oncological control in the follow-up biopsies of 84% and 76% of patients, respectively.^(6)^

In addition to these effective midterm oncological results, IRE offers an attractive safety profile with no high-grade adverse events, a major advance in comparison with radical treatments and other thermal ablative techniques.^(5,6)^

The limitations of this technique include its use in patients with severe arrhythmia, the high cost inherent to the technique (especially in developing countries), and the need for general anesthesia.^(7,8)^ Additionally, it requires interventional radiologists with high expertise in ablative methods and meticulous needle positioning.

## CONCLUSION

Irreversible electroporation is a safe and promising option and should be considered during treatment planning for patients with prostate cancer.
